# Heterogeneous Systemic IgG Responses to *Porphyromonas gingivalis* Gingipains in Advanced Periodontitis

**DOI:** 10.1002/cre2.70391

**Published:** 2026-06-16

**Authors:** Steffen Büttner, Sonja H. M. Derman, Alexander Simonis, Alicia M. Meyer‐Hofmann, Max von Kohout, Manuel Koch, Anna Greta Barbe

**Affiliations:** ^1^ Faculty of Medicine and University Hospital Cologne, Polyclinic for Operative Dentistry and Periodontology University of Cologne Cologne Germany; ^2^ Department I of Internal Medicine Faculty of Medicine and University Hospital Cologne University of Cologne Cologne Germany; ^3^ Faculty of Medicine and University Hospital Cologne Center for Molecular Medicine Cologne University of Cologne Cologne Germany; ^4^ German Center for Infection Research (DZIF), Partner Site Bonn‐Cologne Cologne Germany; ^5^ Institute for Medical Statistics and Computational Biology University of Cologne Cologne Germany; ^6^ Faculty of Medicine and University Hospital Cologne Institute for Dental Research and Oral Musculoskeletal Biology University of Cologne Cologne Germany; ^7^ Faculty of Medicine and University Hospital Cologne Center for Dental, Oral and Maxillofacial Medicine (Central Facilities) University of Cologne Cologne Germany

**Keywords:** gingipains, host immune response, immunoglobulin G, periodontitis, *Porphyromonas gingivalis*, systemic inflammation

## Abstract

**Objectives:**

To evaluate serum IgG responses against *Porphyromonas gingivalis* gingipains RgpB and Kgp in patients with stage III and IV periodontitis and examine associations with clinical periodontal parameters.

**Materials and Methods:**

This exploratory cross‐sectional study included participants with advanced periodontitis (Stage III and IV). Clinical periodontal parameters (number of teeth, bleeding on probing in percent [BoP%], probing pocket depth [PPD], bone loss‐to‐age ratio, periodontal inflamed surface area [PISA] per tooth) were recorded. Blood samples were collected and serum IgG responses against recombinant RgpB and Kgp were analyzed. Serum IgG signals were quantified using enzyme‐linked immunosorbent assay (ELISA).

**Results:**

All 42 participants (mean age 62 years) exhibited measurable serum IgG responses against *P. gingivalis* gingipains. IgG signals (optical density) ranged from 0.13 to 1.46 for RgpB and 0.34 to 1.51 for Kgp. RgpB‐specific IgG levels were higher in Stage IV versus Stage III periodontitis (*p* = 0.023), whereas Kgp‐specific IgG levels showed no significant difference between stages (*p* = 0.249). Anti‐RgpB IgG levels correlated positively with age (*ρ* = 0.362, *p* = 0.019) and inversely with the number of teeth (*ρ* = −0.372, *p* = 0.015), whereas anti‐Kgp IgG showed no statistically significant correlations with the assessed clinical parameters.

**Conclusions:**

Serum IgG binding signals to RgpB and Kgp were measurable by ELISA and showed substantial heterogeneity among patients with advanced periodontitis, with considerable overlap between disease stages. Anti‐RgpB IgG was associated with age and tooth loss, whereas inflammatory measures showed no significant correlations and anti‐Kgp IgG showed only weak trend‐level associations. Overall, total gingipain‐specific IgG appears to reflect cumulative periodontal disease burden and exposure history more clearly than current inflammatory activity in this cohort, with limited value as a standalone marker for differentiating Stage III and Stage IV periodontitis.

## 1 Introduction

Periodontitis is a chronic inflammatory disease characterized by progressive destruction of the tooth‐supporting periodontium (Page and Kornman 1997), and represents one of the most prevalent inflammatory conditions worldwide (Hashim et al. 2025). Its development is driven by the shift from a symbiotic to dysbiotic subgingival microbiome that triggers a sustained host immune response, ultimately leading to connective tissue degradation and alveolar bone loss (Meyle and Chapple 2015; Hajishengallis 2015). Within this dysbiotic microbial community, specific periodontal pathogens play key roles in modulating host inflammatory pathways, initially manifesting as reversible inflammation of the gingiva (Löe et al. 1965). If left untreated, the persistent activation of both the innate and adaptive immune systems can result in irreversible destruction of the supporting structures of the teeth, leading to periodontitis of varying severity (Papapanou et al. 2018).

*Porphyromonas gingivalis* plays a pivotal role in the development of periodontitis, regardless of its abundance within the biofilm (Rafiei and Sayehmiri 2018). The Gram‐negative, obligate anaerobic bacterium colonizes the subgingival environment and has developed multiple strategies to evade immune surveillance and persist in inflammatory conditions (How et al. 2016). As a keystone pathogen, *P. gingivalis* is capable of inducing microbial dysbiosis and promoting chronic inflammatory tissue destruction by modulating the host immune response (Hajishengallis et al. 2012). It produces a variety of virulence factors that contribute to its ability to colonize host tissues, evade immune responses and promote inflammation. These include fimbriae for adhesion, a capsule for immune evasion, lipopolysaccharides that modulate host signaling, and outer membrane vesicles involved in host‐pathogen interactions (Xu 2020).

In addition, *P. gingivalis* produces gingipains, a family of cysteine proteases with trypsin‐like activity that includes the arginine‐specific RgpA and RgpB and the lysine‐specific Kgp (Xu 2020). Structurally, RgpA and Kgp consist of a catalytic domains that mediate adhesion and coaggregation with other oral bacteria, followed by a C‐terminal that contains a hemagglutinin‐adhesin domain. In contrast, RgpB possesses the catalytic domain and immunoglobulin‐like domain like both of the other gingipains, but does not contain the COOH‐terminal hemagglutinin domain (Guo et al. 2010). These enzymes account for approximately 85% of the extracellular proteolytic activity of *P. gingivalis* and play a critical role in tissue destruction (Potempa et al. 1997). Their ability to contribute to immune dysregulation and inflammation by activating host matrix metalloproteinases, inactivating immune inhibitors, degrading immune components, and cleaving immune cell receptors makes them key contributors to the pathogenicity of the bacterium (Guo et al. 2010; Banbula et al. 2000; Tada et al. 2003). In addition to these roles, gingipains facilitate biofilm development and coaggregation with other oral pathogens, thereby strengthening polymicrobial survival within the subgingival niche (Bao et al. 2014; Haraguchi et al. 2014). They are also required for fimbrial maturation, with RgpB essential for FimA processing and both RgpA and RgpB contributing to Mfa1 polymerization (Karin Kristoffersen et al. 2015; Lee et al. 2018). By degrading fibrinogen and heme proteins such as hemoglobin and haptoglobin, gingipains further secure critical nutrients for bacterial growth (Sroka et al. 2001). At the same time, their ability to cleave complement factors C3, C4, and C5 not only prevents bacterial clearance but also enhances inflammation through the release of C5a (Potempa et al. 2008; Popadiak et al. 2007). Collectively, these mechanisms underline the designation of gingipains as main virulence factors, linking bacterial persistence with chronic tissue destruction and immune dysregulation.

Recent work supports gingipains as candidate serological markers of periodontal activity. Anti‐Rgp IgG is elevated in periodontitis and aligns with inflammation and alveolar bone loss, while overall case‐control discrimination is modest. However, Kgp‐specific responses, application of the 2018 periodontal staging scheme (Papapanou et al. 2018), and variable importance across granular clinical metrics were not addressed (Kindstedt et al. 2024). Antibody responses against other *P. gingivalis* virulence factors, such as FimA, have also been found to be increased in individuals with type 2 diabetes mellitus, suggesting that systemic comorbidities may amplify humoral responses against *P. gingivalis* (Groeger et al. 2025).

Despite increasing interest in gingipains as immunologically relevant virulence factors of *P. gingivalis*, the extent to which systemic antibody responses to these enzymes reflect periodontal disease severity or broader host–pathogen interactions remains incompletely understood. In particular, it is unclear whether gingipain‐specific IgG responses reflect current periodontal inflammatory activity, cumulative disease exposure, or heterogeneous host immune responses to microbial challenge. Therefore, our exploratory study in individuals with advanced periodontitis aimed to characterize serum IgG responses to the gingipains RgpB and Kgp and explore their associations with clinical periodontal parameters reflecting inflammatory activity and cumulative tissue destruction.

## 2 Materials and Methods

### 2.1 Study Design

This explorative cross‐sectional study was conducted at the Department for Operative Dentistry and Periodontology, University Hospital Cologne, Germany. Consecutive recruitment of participants with periodontitis and data collection took place from March 2024 to March 2025. A small convenience reference set of periodontally healthy volunteers (*n* = 5) was also included to verify assay performance and provide a descriptive reference range. This reference set was not intended or powered for formal case‐control comparisons. The study was approved by the Ethics Committee of the Medical Faculty, University of Cologne (approval number: 23‐1158_1), prospectively registered in the German Clinical Trials Register (DRKS00032671), and complied with the Declaration of Helsinki. All participants provided written informed consent.

### 2.2 Inclusion and Exclusion Criteria

Participants were eligible for inclusion if they were ≥ 18 years of age. Only patients with severe periodontitis (defined as Stage III or IV and Grade B or C) were enrolled, as well as periodontally healthy reference individuals (max PPD ≤ 3 mm) identified during routine diagnostic visits. Severe periodontitis cases were selected to focus on clinically significant disease and to ensure a homogeneous sample.

Exclusion criteria included any medical condition or circumstance that contraindicated venipuncture, such as known coagulopathies, recent blood donations, acute infections or fever, surgical procedures within the preceding 7 days, or generally poor health status.

### 2.3 Study Procedures

All participants attended a single study visit. To ensure technical comparability, the same sampling, processing, and storage procedures were performed in all participants, and all sera were analyzed in the same assay runs. On the day of inclusion, venous blood was drawn, processed the same day, and stored at −80°C. A full periodontal chart was recorded on that day to ensure temporal alignment with serum sampling. Case assignment followed the 2018 Classification (Stage III–IV) (Papapanou et al. 2018). For new patients, radiographs were obtained for periodontal staging; for patients already in periodontal care, the existing diagnosis and radiographs were used and only the current clinical status was re‐evaluated. Relevant eligibility criteria and detailed sampling/processing parameters are described below.

### 2.4 Population and Clinical Data Collection

Demographic and general health data were collected, including age, sex, the number of systemic conditions, and diabetes status. Periodontal examinations were performed at six sites per tooth. Recorded variables included the number of remaining teeth, reflecting cumulative tooth loss as an indicator of disease severity. Bleeding on probing (BoP, calculated as a percentage) was assessed at each site (Lang et al. 1986). The probing pocket depth (PPD, mm), clinical attachment loss (CAL, mm) and radiographic bone loss adjusted for age were also documented (Tonetti and Claffey 2005). The periodontal inflamed surface area (PISA) was modified and calculated as PISA per tooth (mm^2^) to enable statistical comparability regardless of the influence of the number of teeth on this parameter. For this purpose, the PISA value was divided by the number of teeth (Nesse et al. 2008).

### 2.5 Blood Sampling

Venous blood samples (2 × 10 mL) were collected from all participants at inclusion and centrifuged (2000 rpm × *g*) for 10 min. The supernatant was transferred in 1 mL aliquots into 1.5 mL microcentrifuge tubes and incubated at 56°C for 30 min in a heat block to inactivate complement. After incubation, samples were centrifuged (17000 rpm × *g*) for 1 min. The resulting supernatant was transferred into labeled microcentrifuge tubes (1 mL per tube). All serum samples were stored at −80°C until further analysis (Simonis et al. 2023).

### 2.6 Protein Purification

Recombinant Arg‐gingipain (RgpB) and Lys‐gingipain (Kgp) of *P. gingivalis* ATCC 33277 strain were expressed with an N‐terminal Twin‐Strep‐tag in *Escherichia coli* BL21 (DE3) using a modified protocol based on previously published approaches (Margetts et al. 2000; Veillard et al. 2015). Purity was verified by SDS‐PAGE while protein concentrations were determined spectrophotometrically before storage at −20°C. Detailed cloning, culture and buffer conditions are provided in the Supporting Information S1: Appendix (Margetts et al. 2000; Veillard et al. 2015).

### 2.7 Antibody Measurements

Serum IgG against RgpB and Kgp was quantified by indirect enzyme‐linked immunosorbent assay (ELISA). Plates were coated with antigen, blocked with bovine serum albumin buffer, incubated with 1:100 diluted serum and horseradish peroxidase‐conjugated anti‐human IgG, developed with tetramethylbenzidine, and read at 450 nm. Antibody levels were reported as blank‐corrected optical density (OD) 450 values from triplicate measurements. Full assay conditions are described in the Supporting Information S1: Appendix.

### 2.8 Statistical Methods

All data were analyzed using SPSS software (IBM Corp., Armonk, NY, USA; Version 29) and R for visualizations. Descriptive statistics were calculated for demographic, clinical and serological parameters. Continuous variables are presented as mean ± standard deviation and range, while categorical variables are shown as absolute and relative frequencies. Group differences between Stage III and Stage IV periodontitis patients were analyzed using the Mann–Whitney *U* test for continuous variables and Fisher's exact test for categorical variables. Healthy references were shown descriptively and were not included in inferential group comparisons. Correlations between antibody levels and clinical parameters were analyzed using Spearman rank correlation coefficients. To identify key predictors while accounting for model selection uncertainty, we employed a multi‐model inference approach determining which predictors contribute robustly across multiple specifications rather than selecting a single best model (Burnham and Anderson 2004). To curb overfitting, we restricted models to ≤ 3 of 7 periodontal predictors, yielding 63 candidates per outcome plus an intercept‐only null. The change in corrected Akaike Information Criterion (ΔAICc) quantified predictor informativeness relative to the null, and models were ranked using AICc. Variable importance was calculated as the sum of Akaike weights across models containing each predictor, normalized to 100%. Variance inflation factors (VIF) indicated elevated collinearity between BoP% and PISA per tooth (VIF > 5), suggesting shared inflammatory information. A two‐sided significance level of *p* < 0.05 was considered statistically significant.

## 3 Results

### 3.1 Baseline Clinical and Periodontal Characteristics

Forty‐two periodontitis patients were included (Stage III, *n* = 17; Stage IV, *n* = 25). Baseline characteristics are outlined in Table 1 (along with those for healthy references). Their mean age was 62.07 ± 13.46 years, with an equal distribution between males and females. The majority were non‐smokers (most smokers were in the Stage IV group), and a significantly higher proportion of Stage III versus Stage IV patients had more of their own teeth (mean 25.9 ± 3.3 vs. 21.6 ± 5.5; *p* = 0.003). All participants had generalized periodontitis; although periodontal parameters tended to be worse in the Stage IV group, the only significant difference was in the bone loss‐age ratio (0.77 in Stage III vs. 1.11 in Stage IV; *p* = 0.008).

**Table 1:** Baseline demographic and health‐related characteristics of Stage III and Stage IV periodontitis patients and healthy references.

Baseline characteristics	Healthy references	Periodontitis patients		*p*‐values
		Stage III	Stage IV	
*N*	5	17	25	N/A
Age, mean ± SD	23.60 ± 1.95	63.06 ± 14.56	61.40 ± 13.22	0.710
Sex, n (%)				
Male	1 (20.0)	9 (52.9)	12 (48.0)	1.000
Smokers, *n* (%)				
Yes	0 (0)	4 (23.5)	11 (44.0)	0.207
Diabetes, *n* (%)				
Yes	0 (0)	5 (29.4)	1 (4.0)	0.032
Number of systemic diseases, mean	0	1.88	1.36	0.160
Periodontal parameters				
Grade B, *n* (%)	N/A	10 (58.8)	11 (44.0)	N/A
Grade C, *n* (%)	N/A	7 (41.2)	14 (56.0)	N/A
Number of teeth, mean ± SD	28 ± 0.00	25.94 ± 3.31	21.64 ± 5.48	0.003
BoP (%), mean ± SD	3.84 ± 2.35	20.74 ± 16.94	25.52 ± 23.02	0.750
Max PPD (mm), mean ± SD	2.80 ± 0.45	7.06 ± 2.16	7.68 ± 1.93	0.190
PISA per tooth (mm^2^), mean ± SD [range]	1.24 ± 0.86 [0.00‐2.28]	15.26 ± 14.14 [0.03–44.36]	22.72 ± 24.01 [1.07–109.01]	0.420
Bone loss‐age ratio, mean ± SD	N/A	0.77 ± 0.29	1.11 ± 0.46	0.008

*Note: p*‐values refer to comparisons between Stage III and Stage IV periodontitis patients. Continuous variables were compared using the Mann–Whitney *U* test. Sex, smoking status, and diabetes were compared using Fisher's exact test. Healthy references are shown descriptively and were not included in inferential group comparisons.

Abbreviations: BoP, bleeding on probing; N/A, not applicable; PISA, periodontal inflamed surface area; PPD, probing pocket depth; SD, standard deviations.

### 3.2 ELISA‐Detected Antibodies

**Figure 1:**
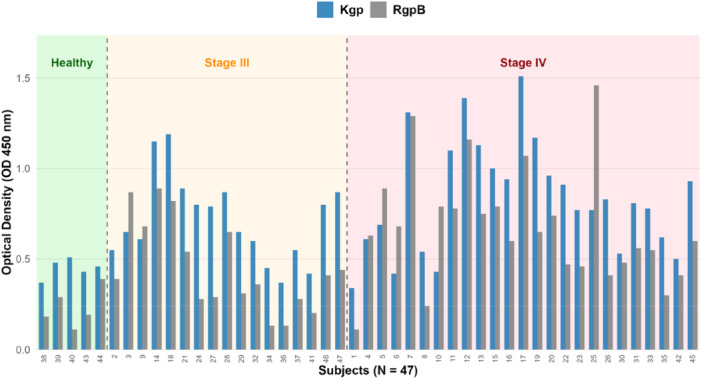
Serum IgG responses against RgpB and Kgp gingipains (optical densities). Serum IgG responses against recombinant RgpB and Kgp were measured by enzyme‐linked immunosorbent assay (ELISA) at a serum dilution of 1:100. Blank‐corrected optical density (OD450) values are shown for all participants. Gray bars represent anti‐RgpB IgG signals, and blue bars represent anti‐Kgp IgG signals.

Serum IgG binding signals against RgpB and Kgp were measurable by ELISA at a 1:100 dilution across the study population (Figure 1). Mean OD values across the total cohort were 0.58 ± 0.31 for RgpB and 0.79 ± 0.29 for Kgp. Mean OD values for Stage III patients were 0.45 ± 0.25 for RgpB and 0.72 ± 0.23 for Kgp; for Stage IV patients, the respective values were 0.67 ± 0.32 and 0.84 ± 0.31 (Table 2).

**Table 2:** Baseline serological characteristics (optical densities, measured by ELISA) of Stage III and Stage IV periodontitis patients and healthy references.

Optical density	Healthy references	Periodontitis patients		*p*‐values
		Stage III	Stage IV	
RgpB, mean ± SD [range]	0.23 ± 0.11 [0.11–0.39]	0.45 ± 0.25 [0.13–0.89]	0.67 ± 0.32 [0.11–1.46]	0.023
Kgp, mean ± SD [range]	0.45 ± 0.05 [0.37–0.51]	0.72 ± 0.23 [0.37–1.19]	0.84 ± 0.31 [0.34–1.51]	0.249

*Note: p*‐values were calculated using the Mann–Whitney *U* test and refer to comparisons between Stage III and Stage IV. Healthy references are shown descriptively and were not included in inferential group comparisons.

Abbreviations: ELISA, enzyme‐linked immunosorbent assay; SD, standard deviation.

### 3.3 Stage‐Related Differences in Antibody Levels

**Figure 2:**
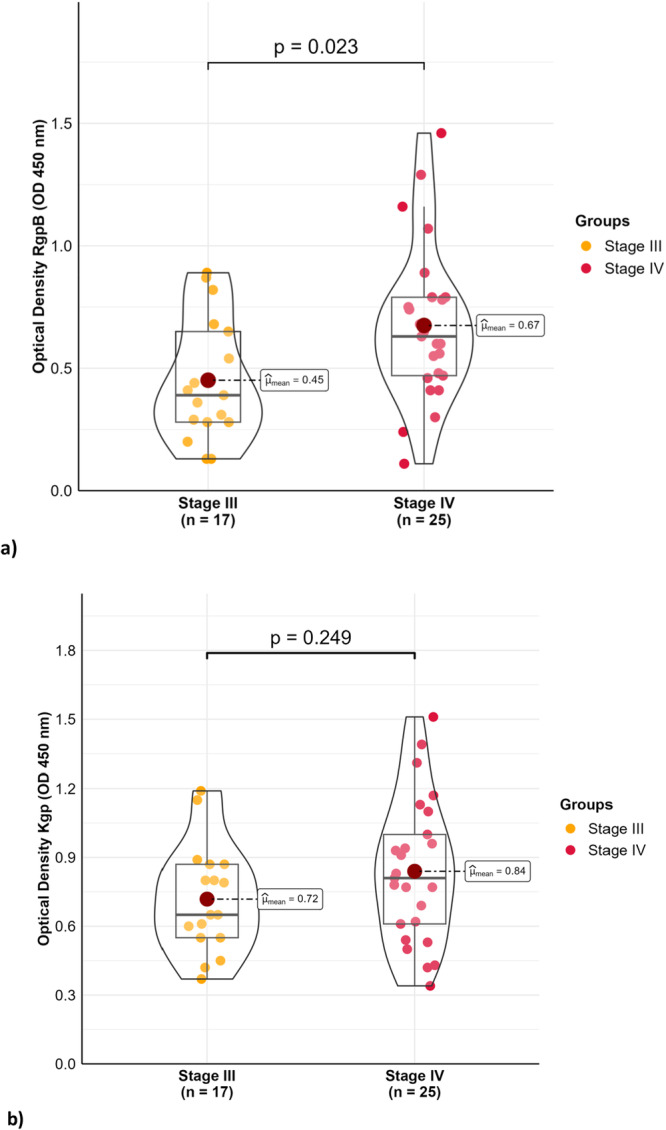
Group differences in (a) anti‐RgpB responses and (b) anti‐Kgp IgG responses. Anti‐RgpB IgG (a) and anti‐Kgp IgG (b) responses were measured by ELISA at a serum dilution of 1:100 and are shown as blank‐corrected OD450 values. Each point represents one periodontitis patient. Violin plots show the distribution of values, boxes indicate the interquartile range, horizontal lines indicate the median, and larger dark points with dashed labels indicate group means. *p*‐values indicate Mann–Whitney *U* tests comparing Stage III and Stage IV periodontitis patients.

Comparisons between Stage III and Stage IV periodontitis patients showed higher anti‐RgpB IgG levels in Stage IV than in Stage III patients (*p* = 0.023), whereas anti‐Kgp IgG levels did not differ significantly between stages (*p* = 0.249; Table 2 and Figure 2). Healthy references are shown descriptively and were not included in inferential group comparisons.

### 3.4 Correlations Between Antibody Levels and Clinical Parameters

Correlation analyses within the periodontitis cohort showed that anti‐RgpB IgG was positively correlated with age (Spearman *ρ* = 0.362, *p* = 0.019) and inversely correlated with the number of teeth (*ρ* = −0.372, *p* = 0.015). No significant associations were observed between anti‐RgpB IgG and BoP% (*ρ* = 0.152, *p* = 0.337) or PISA per tooth (*ρ* = 0.108, *p* = 0.498).

For anti‐Kgp IgG, no statistically significant correlations were detected with age (*ρ* = 0.137, *p* = 0.387), number of teeth (*ρ* = 0.030, *p* = 0.851), BoP% (*ρ* = 0.265, *p* = 0.090), or PISA per tooth (*ρ* = 0.264, *p* = 0.091), although trend‐level positive associations were observed for BoP% and PISA per tooth (Table 3).

**Table 3:** Correlation of clinical parameters with anti‐RgpB and anti‐Kgp IgG levels.

Periodontitis patients	Anti‐RgpB IgG level		Anti‐Kgp IgG level	
	*ρ*	*p*‐value	*ρ*	*p*‐value
Age	0.362	0.019	0.137	0.387
Number of teeth	−0.372	0.015	0.030	0.851
BoP%	0.152	0.337	0.265	0.090
PISA per tooth	0.108	0.498	0.264	0.091

*Note:* Spearman rank correlation coefficients (*ρ*) and corresponding two‐sided‐sided *p*‐values are shown for all clinical parameters.

Abbreviations: BoP, bleeding on probing; PISA, periodontal inflamed surface area.

### 3.5 Variable Importance Model for RgpB and Kgp

To further explore which clinical and demographic variables contributed most consistently to gingipain‐specific IgG signals, we applied the exploratory multi‐model inference approach described in Section 2.8. This analysis assessed the relative consistency of predefined predictors across AICc‐ranked candidate models. Variable importance was calculated from summed Akaike weights and is therefore reported as relative predictor importance rather than as *p*‐values or evidence of statistical significance.

**Figure 3:**
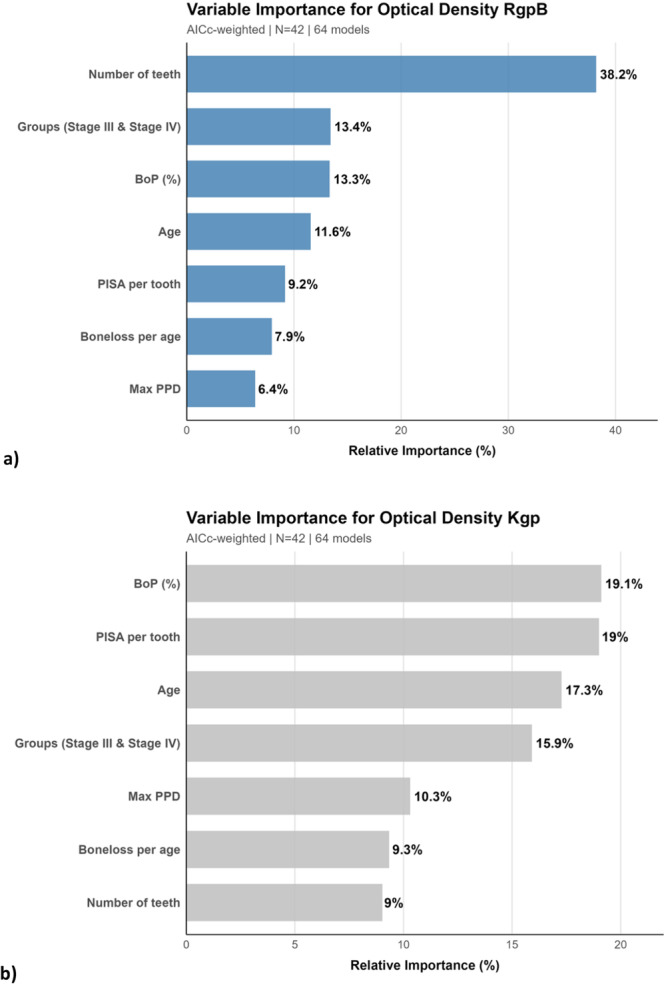
Relative importance of demographic and periodontal predictors for (a) anti‐RgpB IgG and (b) anti‐Kgp IgG signals. Relative predictor importance for anti‐RgpB IgG (a) and anti‐Kgp IgG (b) was derived from multi‐model inference across candidate regression models. Bars represent normalized sums of Akaike weights across all models containing the respective predictor. The intercept‐only model was included as the null model.

For anti‐RgpB, the number of teeth dominated (38.2%), followed by Groups (13.4%) and BoP% (13.3%), with the mean max. PD showing the lowest contribution (6.4%) (Figure 3a). For anti‐Kgp, the highest relative importance was observed for BoP% (19.1%), PISA per tooth (19.0%), and age (17.3%), while number of remaining teeth contributed least (9.0%) (Figure 3b).

## 4 Discussion

Systemic antibody responses to gingipains appear to represent heterogeneous host response signatures of exposure to *P. gingivalis* in patients with periodontitis. In this cohort, serum IgG responses to RgpB and Kgp showed substantial inter‐individual variability and did not follow a uniform association pattern. Anti‐RgpB IgG was positively associated with age and inversely associated with the number of remaining teeth, supporting the interpretation that RgpB‐specific systemic IgG may partly reflect cumulative exposure and accumulated periodontal damage. In contrast, anti‐RgpB IgG was not associated with current inflammatory measures such as BoP% or PISA per tooth. Anti‐Kgp IgG showed no statistically significant correlations with the assessed clinical parameters, although positive trends were observed for BoP% and PISA per tooth. These findings indicate that, in this cohort, gingipain‐specific IgG responses were not consistently aligned with current periodontal inflammatory measures. This supports a cautious interpretation of systemic gingipain‐specific IgG as a heterogeneous serological readout that may be influenced by cumulative disease experience, microbial exposure history, and individual host response patterns (Ebersole et al. 2020; Papapanou et al. 2004).

The demographic and baseline characteristics of our study population reflect established epidemiological patterns of periodontitis. The age distribution of the cohort, dominated by older adults with advanced disease, aligns with population‐based data from the Sixth German Oral Health Study and NHANES, which demonstrate a strong age‐related increase in periodontitis prevalence and severity, with advanced periodontal disease occurring more frequently in older individuals (Eickholz 2025; Eke et al. 2012, 2018).

In this cohort, ELISA analyses demonstrated measurable IgG responses against both RgpB and Kgp across all groups, with descriptively higher mean OD values for Kgp. As no formal direct statistical comparison between the two antigens was performed, these differences should be interpreted as antigen‐specific ELISA signal patterns rather than as directly equivalent quantitative antibody concentrations. Nevertheless, the observed pattern may partly reflect structural differences between the enzymes. Both RgpA and Kgp contain hemagglutinin/adhesin domains that contribute to antigenicity, whereas RgpB consists of a catalytic and immunoglobulin‐like domain and lacks these additional adhesin regions, which may reduce its immunogenic potential (Guo et al. 2010; O'Brien‐Simpson et al. 2009; Potempa et al. 1995). Strain‐specific differences may also influence systemic antibody responses. For example, certain clinical isolates of *P. gingivalis* (e.g., A7436) upregulate RgpB expression under heme‐ and iron‐limiting conditions, potentially reducing systemic exposure, whereas the laboratory strain ATCC 33277 used in this study does not exhibit this response (Śmiga et al. 2024). A few individuals (notably subjects 7 and 25) exhibited inverted antibody patterns, with higher anti‐RgpB than anti‐Kgp IgG levels. Such variability is consistent with previous reports of heterogenous human antibody responses to *P. gingivalis*, reflecting differences in host immune recognition, microbial strain diversity, and exposure history (Ebersole et al. 2020).

Age‐dependent differences and heterogeneity of systemic antibody responses to the whole bacterium in periodontitis populations have been reported previously, supporting the concept that humoral readouts are shaped by complex host and exposure factors rather than single clinical indices (De Nardin et al. 1991). Importantly, earlier studies that combined serology with subgingival microbiological assessment demonstrated direct relationships between serum anti‐*P. gingivalis* IgG levels and the amount of subgingival *P. gingivalis*, also showing strong links between *P. gingivalis* presence and BoP at sampled sites (Kojima et al. 1997). More recently (in the PerioGene North cohort), elevated anti‐Rgp IgG identified a subset of periodontitis cases characterized by high BoP and advanced alveolar bone loss, supporting a link between gingipain‐directed systemic immunity and destructive periodontal phenotypes (Kindstedt et al. 2024). These findings could not be confirmed in our cohort, however, suggesting that RgpB‐specific serum IgG may not be closely aligned to concurrent local inflammatory activity but rather reflects inter‐individual differences in exposure, colonization dynamics, and host immune responsiveness. For anti‐Kgp IgG, only trend‐level associations with inflammatory measures were observed in our small cohort. As subgingival *P. gingivalis* and biofilm composition were not assessed, the readout remains indirect and severe destruction may also occur in polymicrobial communities not dominated by *P. gingivalis*.

Comparisons between clinical stages revealed only modest differences. Anti‐RgpB IgG was higher in Stage IV than in Stage III, whereas anti‐Kgp IgG did not differ between stages, with substantial overlap of individual values across both groups. The higher anti‐RgpB IgG levels in Stage IV should be interpreted in the context of cumulative periodontal disease burden, as Stage IV is partly characterized by advanced tissue destruction and tooth loss. Accordingly, the inverse association between anti‐RgpB IgG and the number of remaining teeth supports the interpretation that anti‐RgpB IgG may reflect accumulated periodontal damage and exposure history rather than stage category alone. Similar limited stage separation for antibodies against recombinant gingipains has been reported previously, supporting the limited utility of recombinant gingipain antibodies for stratifying periodontal disease categories (Massarenti et al. 2025). Several factors may explain these attenuated group differences, including pronounced inter‐individual heterogeneity of anti‐*P. gingivalis* serological profiles, which can obscure stage‐related signals in cross‐sectional cohorts (Ebersole et al. 2020). In addition, advanced periodontitis can occur in polymicrobial dysbiosis without *P. gingivalis* dominance, plausibly resulting in low gingipain‐specific titers in some severe cases (Griffen et al. 1998). Prior studies of anti‐*P. gingivalis* IgG responses suggest subclass‐specific patterns with IgG2 are often more informative than IgG4, which may be masked when measuring total IgG only—which could mean that subclass composition may matter (Whitney et al. 1992; Polak et al. 1995).

The exploratory variable‐importance analysis further suggested that anti‐RgpB and anti‐Kgp IgG may capture partly different aspects of host–pathogen interaction. For anti‐RgpB IgG, the number of remaining teeth showed the highest relative importance, consistent with the inverse correlation analysis and compatible with an interpretation of anti‐RgpB IgG as a readout related to accumulated periodontal destruction. For anti‐Kgp IgG, relative importance was more evenly distributed, with inflammatory measures such as BoP% and PISA per tooth contributing more prominently than tooth count. This pattern may be biologically plausible, as Kgp is a multidomain lysine‐specific protease involved in hemoglobin binding, heme acquisition, and interactions with host proteins, processes that may be particularly relevant in inflamed and bleeding periodontal pockets (de Diego et al. 2014). However, these findings should be interpreted cautiously, as variable‐importance values indicate relative predictor consistency across candidate models and do not represent *p*‐values or evidence of statistical significance. This is particularly relevant for anti‐Kgp IgG, for which Spearman correlations with inflammatory parameters showed only weak positive trends.

Our exploratory study is limited by the small sample size, which reduces statistical power and increases uncertainty in model‐based variable‐importance estimates. Its cross‐sectional design precludes causal inference, and the restriction to advanced periodontitis limits generalizability to earlier disease stages. Subgingival *P. gingivalis* was not quantified, and the overall subgingival biofilm composition was not profiled. Therefore, the serological signals cannot be directly linked to current pocket colonization or to specific microbial community structures. Antibody measurements were restricted to total IgG. Potential subclass‐specific patterns were not assessed. A small periodontally healthy reference set was included for assay plausibility only and was not intended or powered for inferential case‐control comparisons. Because this reference set was younger and not age‐matched, descriptive differences versus periodontitis patients should be interpreted cautiously. ELISA readouts quantify antigen‐binding reactivity, but do not determine whether these antibodies functionally inhibit gingipain proteolytic activity or otherwise neutralize gingipain‐mediated virulence functions. Given unequal tooth counts at a single visit, we normalized PISA as PISA per tooth, which is a statistical convenience rather than a biologically exact measure, as it does not account for differences in root surface area across tooth groups and may yield different values when the same inflamed area is distributed across fewer versus more teeth.

## 5 Conclusions

Serum IgG binding signals to RgpB and Kgp were measurable by ELISA across the study population, including patients with advanced periodontitis and the small healthy reference set, without defining a threshold for seropositivity. Among patients with advanced periodontitis, these signals showed substantial inter‐individual variability and considerable overlap between disease stages. The observed associations of anti‐RgpB IgG with age and tooth loss, together with the lack of significant correlations with inflammatory measures and only weak trend‐level associations for anti‐Kgp IgG, suggest that systemic gingipain‐specific IgG responses may reflect cumulative periodontal disease burden and exposure history more clearly than current inflammatory activity in this cohort. Consequently, total gingipain‐specific IgG appears to have limited value as a standalone serological marker for differentiating Stage III and Stage IV periodontitis. Future studies should explore whether more refined serological approaches (including IgG subclass analysis, functional antibody assays, integration with microbial detection methods, larger cohorts, and age‐matched healthy controls) can better capture biologically meaningful host–pathogen interactions in periodontal disease.

## Author Contributions

**Steffen Büttner:** conceptualization, formal analysis, investigation, resources, data curation, writing – original draft. **Alexander Simonis:** methodology, investigation, writing – review and editing, supervision. **Alicia M. Meyer‐Hofmann:** writing – review and editing. **Max von Kohout:** statistics, writing – review and editing. **Sonja H. M. Derman:** conceptualization, validation, writing – review and editing, supervision. **Manuel Koch:** methodology, validation, resources, writing – review and editing, supervision. **Anna Greta Barbe:** conceptualization, methodology, resources, writing – review and editing, supervision, project administration.

## Funding

The authors have nothing to report.

## Ethics Statement

The study received ethical approval from the local ethics review board of the University of Cologne, Cologne, Germany (approval number: 23‐1158_1) and was prospectively registered in the German Clinical Trials Register (DRKS00032671). All procedures complied with the ethical standards of the institutional research committee, the 1996 Helsinki Declaration, and subsequent amendments or comparable ethical standards.

## Consent

All participants provided written informed consent.

## Conflicts of Interest

The authors declare no conflicts of interest.

## Supporting information

Supporting File

## Data Availability

The data that support the findings of this study are available from the corresponding author upon reasonable request (S. Büttner, steffen. buettner1@uk-koeln.de).
