# Supplementary material for: Heterogeneous Systemic IgG Responses to Porphyromonas gingivalis Gingipains in Advanced Periodontitis
Source: Clin Exp Dent Res. 2026 Jun 16;12(3):e70391. doi: 10.1002/cre2.70391 (PMC13271254; doi:10.1002/cre2.70391)
Supplement: Supplementary file 1 — Supporting File [file CRE2-12-e70391-s001.docx]

**Appendix**

**Protein purification**

Recombinant Arg-gingipain (RgpB; protease region WP 281123075, aa: 229-493) and Lys-gingipain (Kgp; protease region 1CVR A, aa: 2-430) of *P. gingivalis* ATCC 33277 strain were expressed with a N-terminal Twin-Strep-tag in *Escherichia coli* BL21 (DE3) using a modified protocol based on previously published approaches.(Margetts et al., 2000; Veillard et al., 2015) The two genes were amplified by PCR and cloned via Nhel and BamHI into a modified Pet28 (+) (NovoPro Bioscience Inc.) vector containing an N-terminal Twin-Strep-tag. After sequence verification, the Eschericha coli BL21 (DE3) were transformed with the two plasmids and overnight precultures were grown in LB medium supplemented with kanamycin (50 μg/mL) at 37 °C. Main cultures were inoculated into NZY auto-induction medium containing kanamycin and cultivated in Ultra Yield flasks at 32 °C with shaking (220 rpm) for 20-24 h. Cells were harvested by centrifugation (3000 rpm, 15 min, 4 °C), washed, and stored at -20 °C. Pellets were resuspended in lysis buffer (50 mM Tris-HCL, pH 8.0, 0.5 M NaCl, 0.5% Tween-20) and disrupted by sonication (5 cycles of 1 min, 3 s on/1 s off, 27% amplitude). Cell debris was removed by centrifugation at 18,000 rpm for 40 min at 4 °C. The clarified lysate was applied to Strep-TactinXT 4Flow agarose (IBA) pre-equilibrated with binding buffer (50 mM Tris-HCl, 1mM EDTA, 150 mM NaCl, pH 8.0). After extensive washing with the binding buffer, proteins were eluted with biotin (10 mM biotin in 100 mM Tris-HCL, pH 8.0, 150 mM NaCl). Fractions containing recombinant Arg- or Lys-gingipain were analysed by SDS-Page. Protein concentrations were determined spectrophotometrically at 280 nm, and purified proteins were aliquoted and stored at -20°C until use.

**Antibody measurements**

A total of 0.5 μg of the target protein (RgpB and Kgp) diluted in 50 μL of TBS was coated onto each well of a 96-well microtiter plate and incubated overnight at 4°C. After incubation, wells were washed to remove unbound protein. To block nonspecific binding sites, 100 μL of 3% BSA in TBS was added to each well and incubated for 1.5 hours at room temperature. Simultaneously, serum samples containing potential ligands were diluted 1:100 in 2-3% BSA/TBS. After another washing step, 50μL of the diluted ligand was added to each well and incubated for 1 hour at room temperature. Each serum sample was tested in triplicate. The Rabbit anti-Human IgG Secondary Antibody (Thermo Fisher Scientific, Waltham, MA, USA) conjugate was prepared in 2-3% BSA/TBS in a 1:3000 dilution. Following three additional washing steps, 50 μL of the HRP-conjugated detection reagent was added per well and incubated for another 1 hour at room temperature. After washing 50 μL of TMB (Thermo Fisher Scientific, Waltham, MA, USA) substrate solution was added to initiate to colorimetric reaction. The reaction was stopped after 5 min 45 sec by adding 50 μL of 10% H_2_SO_4_ to each well. Finally, absorbance was measured at 450nm using a Tecan microplate reader. Final antibody levels were calculated as blank-corrected optical density values, obtained by subtracting the mean OD of negative control wells without serum from the raw sample OD.
